# Traité Theoretique et Pratique d'Auscultation Obstetricale

**Published:** 1847-10-01

**Authors:** 


Traite Theorique et Pratique d'Auscultation Obstetricale. Par *J. A. H. Depaul,* D.M., &c. &c. Octavo, pp. 400. Paris, 1847. Labé.

The subject of Obstetrical Auscultation has not attracted that notice and attention in this country which its importance demands. With the exception of Dr. Evory Kennedy, whose admirable volume contributed so much to diffuse a knowledge of it at the time of its publication, 1833, our obstetrical physicians have done but little to advance this most interesting branch of professional research, and, in this respect, have been greatly behind their brethren in France and Germany. The volume before us contains the results of our author's observations commenced ten years ago in the wards of the Maternity Hospital at Paris, under the guidance, we believe, of Professor Dubois, and steadily persevered in ever since both in hospital and in private practice. The author writes as one who is thoroughly acquainted with his subject, and his work is therefore likely to become, we should think, a manual of information on the subject of which it treats.

It is divided into two parts, the Historical and the Didactic. The first, occupying a third of the volume, gives an excellent account of the various treatises and papers which have been published both in France and elsewhere on the subject since the appearance of the admirable memoir of M. Kergaradec—unquestionably the true founder of obstetrical auscultation, although M. Mayor of Geneva had anticipated him in the discovery of one of its principal phenomena—in 1822 down to the present time, with an analytic summary of the contents of the most valuable of these writings. To the intelligent reader this account will prove acceptable; as it is not less instructive than interesting to trace the successive steps by which any branch of useful knowledge has reached to the status in which it now exists. We must, however, at once pass to the contents of the second or didactic part, which treats of the sounds that are discoverable in the abdomen during pregnancy, and the presence of which is more or less peculiar to, and characteristic of, this condition. There are, however, one or two prefatory observations that should be first briefly alluded to.

Dr. Depaul admits that the auscultation of pregnancy is attended with much more difficulty than that of thoracic diseases, and that skill, however great, in the latter department of diagnostic enquiry will not suffice to ensure speedy success in the former. He has known more than one instance of a diligent student not succeeding in distinctly making out the existence either of the uterine or of the fœtal sound, until after several months' repeated trials. M. Dubois, now one of the best obstetrical auscultators in Paris, confesses that he had to serve a long *apprenticeship* (that is the word he uses himself) to the pursuit, before he acquired anything like confidence in the results of his observations. It is undeniable, however, that, notwithstanding the difficulties, it only requires persevering diligence, and the opportunity of a sufficiently ample experience, to enable any one to overcome them all, and to gain that readiness in discovering the presence or absence of those important signs of the pregnant state, which the sense of hearing can alone discover. Dr. Depaul suggests that there should be a regular class for obstetrical auscultation in every good school of medicine; nor do we see how the available and practical knowledge of it can ever be obtained save in this way. Certain it is, that no one will ever be able to instruct himself by the merely occasional opportunities afforded by private practice.

Besides the most perfect silence in the chamber, there are two or three other circumstances which the auscultator will find well to attend to. The rectum and bladder ought, if possible, to be empty at the time of the examination; for, independently of the sounds which may arise from the contents of these viscera, it is obvious that the distension of the abdomen, so produced, will prevent that ready pliancy and yielding of its parietes to the pressure of the stethoscope which it is often necessary to make, especially in the early months of pregnancy. Dr. Depaul prefers the recumbent to any other position for the purpose of auscultatory examination of the uterus. The thighs ought to be somewhat bent, in order to relax as much as possible the abdominal muscles; in this way only, can the contour of the uterine globe be distinctly traced, and the stethoscope be applied to its lateral regions. The height of the bed, on which the woman lies, is a matter of some consequence to the physician; for, if it be too low, he will soon find that the fatigue of stooping will prevent him from continuing his examination so long as he might otherwise wish; not to mention the headache and confusion of hearing which are apt to be produced thereby. Nothing should intervene between the instrument and the abdomen of the patient, but a soft handkerchief or chemise if this be thin and quite smooth; but in obscure cases it is better to apply the instrument directly to the uncovered skin. The corset, or any other tight article of clothing, must have been previously removed.

Dr. Depaul, it should be here remarked, gives a decided preference to mediate over immediate auscultation in obstetrical examinations. The following passage contains his reasons for this purpose.

"In the first place, immediate auscultation cannot be employed, with any prospect of advantage, in the early months of pregnancy, when the uterus is scarcely above the level of the pelvic entrance. The same will be the case in a somewhat more advanced period, when, although the organ is higher up, there are interposed between it and the abdominal parietes folds of intestine or other intervening substance. The naked ear cannot be employed without bringing a large extent of the abdomen in contact with the side of the face. From this circumstance result, first, the friction-sounds inseparable from so extensive a contact; secondly, (and this remark had not escaped the penetration of Laennec,) in order sufficiently to depress the parts that separate the ear from the uterus, it is necessary to employ a much greater degree of force, the consequence of which is that the physician thereby increases the sounds arising from the contraction of his own muscles; and thirdly, even when the pregnancy is far advanced, and especially when there is a tendency to the anterior obliquity of the uterus, there are certain points of it which cannot be conveniently explored, those, for example, adjoining the inguinal regions............. The great advantages of auscultation with the stethoscope are, that the sounds are thereby rendered more distinct and readily perceptible; that we can more easily determine their limits, as well as distinguish the one from the other; and, lastly, that we can with much greater accuracy appreciate their different shades or degrees in point of intensity or force. This is not because the instrument adds aught to their intensity, but merely because it enables the physician to come within a shorter distance of their origin."

In all the cases in which our author has succeeded in detecting the auscultatory signs of pregnancy in the very early months of pregnancy, it was with the aid of the stethoscope; examination with the naked ear having proved quite ineffectual. He then adds :—

" The pressure which is made with the ear necessarily bears upon a large surface, and this has many inconveniences. With the stethoscope, on the contrary, we act upon a limited point, and avoid all friction-sound. In the great majority of cases, moreover, we can, without effort or danger, remove or push aside the stratum of *liquor amnii* interposed between the fœtus and the uterine parietes. This proposition will be received with doubt by those who are in the habit of believing that the uterus is quite distended with fluid during pregnancy. But this opinion is not correct. When the different evolutions which take place during pregnancy succeed one another with regularity, the uterus remains yielding and compressible at all the stages, and this too usually to a considerable degree. Very near the full period, one or two litres of liquid might still be added to the liquor amnii, before complete distension was produced. In several instances, where women have died near their full period but without being delivered, and where the ovum was in all its integrity, I have ascertained the truth of the assertion now made."

We may observe that the stethoscope which Dr. Depaul always uses is one invented by himself, and which is now, he says, very generally used in Paris for obstetrical auscultation. A figure of it is given in his work. After these preliminary remarks, we now proceed to lay before our readers the pith and marrow of our author's very minute and accurate description of the different auscultatory signs of pregnancy; and this we shall do most correctly and briefly by first giving the conclusions, in which he has himself compressed and embodied the contents of each chapter, and then selecting a few passages from the main text to illustrate some of these conclusions at rather greater length. And first, of that sound which has its seat in the uterus itself, and with which the fœtus has nothing directly to do.

1. The denomination of *uterine souffle* is preferable to any other term that has been proposed.

2. The uterine souffle does not resemble other blowing sounds that are perceptible along the trajet of arteries. Like them, indeed, it is isochronous with the contractions of the left ventricle; but it has characters peculiar to itself.

3. It varies exceedingly in respect of tone and persistance, and also as to the point of the uterus where it exists, &c. &c.

4. It has been demonstrated by incontestable facts that it may be perceived in the middle of the 11th, and even at the end of the 10th, week of pregnancy. In general, however, it cannot be discovered till somewhat later.

5. Its intensity goes on increasing until the end of the 7th month ; after this time it makes but little progress. Allowance must be made for individual differences.

6. It is produced in the arteries of the uterus, and may be heard over every point of the organ that is accessible to the ear or to the stethoscope.

7. It is in the peculiar arrangement of the arterial system of the uterus, and in the modifications to which it is liable from the active movements of the fœtus, that we find the best explanation of its production, its irregularities, intermissions, changes in point of situation, &c.

8. Many physicians have erroneously regarded it as a sure and certain sign of pregnancy. Taken by itself, it has not much more value than the other rational signs of this state: but, in connexion with some others, it gives an importance to them, while at the same time it acquires a very great value itself.

9. It cannot in the present day be denied that a sound, in every respect similar to the uterine souffle, may exist when the enlargement of the uterus is owing to a cause altogether different from pregnancy.

10. The death of the fœtus does not appreciably modify it; *a fortiori*, it does not cease upon this event. We cannot, therefore, have recourse to it with advantage, if our object be to ascertain whether the child lives or not.

11. Neither is it modified by the diseases which may attack the fœtus during the course of intra-uterine life.

12. If it be true that a direct relation generally subsists between the size of the placenta and that of the child, we are not warranted in believing, as some writers would have us do, that we can judge of the strength and development of the latter by the intensity, or by any other character, of the uterine souffle.

13. As it is clearly established that there is no necessary relation between the uterine souffle and the placenta, we can readily understand that the morbid conditions of the latter are not discoverable by any modifications of the former.

14. For the same reason, this sound is incapable of giving us any precise notions as to the exact spot where the placenta is attached; and, if chance has sometimes seemed to warrant the idea in question, innumerable facts to the contrary have abundantly proved its general fallacy.

15. To pretend that, by the aid of this sign, we can form any rational conjecture as to the shape of the placenta, is an opinion altogether contradicted by experience, but which was, in some measure, the forced consequence of the erroneous doctrine that the uterine souffle was necessarily connected with the circulation of the placenta.

16. Neither can the presence of double pregnancies be ever made out by the existence of more souffles than one. Experience proves that two and even three distinct souffles may be met with, when the pregnancy is single; and anatomy, moreover, has shown that, in the majority of cases of twins, there is only one placentary mass.

17. The uterine souffle is of no utility in determining the situation of the child in the womb.

18. Lastly, it follows from all that has been now stated, that this sign is of very limited value in respect of its applications to practice.

With regard to the first proposition, Dr. Depaul remarks that, being convinced that the sound in question is not limited to the seat of the placenta, and that it does not necessarily arise from the circulation through its vessels, he objects to the appellation of *placentary souffle* which has been given to it by many writers, and prefers that of *uterine souffle* first proposed by M.

Dubois. To apply the term *pulsation* to the sound is surely very inaccurate; seeing that there is a total absence of any impulse or beat accompanying it. It is a something to be heard, not to be felt. It has usually the character of a soft intermitting puff; at other times it resembles the whistling of the wind through an ill-closed window, only that it is *saccadé* and not continued; while in other cases it is of a graver note, and is not unlike the vibrations of a bass string, or the cooing murmur of a dove. But, whatever be the character of the sound, it is invariably isochronous with the pulse of the mother, and never with that of the fœtus.

There is no point of the uterine globe accessible to the stethoscope, in which this sound *may* not *be* heard. It is, however, over the lateral regions of the womb that it is usually most readily perceptible; but Dr. Depaul is not prepared to say whether it be more frequently present on the right, or on the left, side. Hohl says on the former; Naegele on the latter, admitting, however, that he has found it on both sides at the same time, and occasionally over every part of the uterus. Out of 295 cases in which pregnancy exceeded the fifth month, our author heard a distinct souffle on each side of the uterus, at a little distance from the crural arch in 182. It was discoverable on one side only in 27, and over the fundus of the organ in 43. In 18, the stethoscope could not be placed over any point of the uterus without the sound being readily met with; and, in 12, it was audible at three distinct points, viz. the fundus, and the parts immediately above the crural arch on each side. When the pregnancy was less advanced, Dr. Depaul has given the following results of his experience. In 4 cases where pregnancy had not exceeded the end of the 3rd month, the souffle was heard in the median line above the pubis. In 13 cases, in which pregnancy had advanced another month, it occupied the same region: the stethoscope, it should be stated, required to be pushed somewhat forcibly towards the pelvis to discover it. In 3 cases, in which gestation was about the same period, it was heard on each side. Lastly, in 16 out of the 27 cases, in which pregnancy had reached the end of the fifth month, the sound was perceptible over every accessible part of the uterus; but it was nevertheless easy to determine that it proceeded from the lateral regions.

By the fourth proposition, the reader will perceive that Dr. Depaul confirms the accuracy of Dr. E. Kennedy's assertion that, the uterine souffle *may be* heard before the end of the third month, a period considerably earlier than that which has been fixed by most writers. The usual belief is, that it is not discoverable before the end of the fourth month; and, as a general remark, this is doubtless quite true.

M. Bouillaud and one or two other writers have, it is well known, attributed the blowing sound of pregnancy to the pressure of the enlarged womb upon the iliac arteries, and the consequent partial obstruction to the easy flow of blood through them. On this point our author thus comments:—

"Although I entirely repudiate the doctrine in question, I am ready to admit the possibility of the production of a blowing sound from the cause mentioned, having met with several examples of the kind in my own practice. In these cases, the phenomenon exists also on one of the sides of the uterus; I have never met with it on more than one side in any single case; and, in general, it is upon that which corresponds with the lateral inclination of the organ, when it does exist. It is, like the genuine uterine souffle, completely isochronous with the maternal pulse, and, like it, follows all its changes; but it presents a constant character which will readily serve to distinguish the one from the other. We have now no longer a souffle without pulsation; on the contrary, what is present is a pulsation accompanied by a more or less intense souffle; but I have never met with this last so extended as to render the perception of the first at all difficult. Moreover, while the position of the woman, and consequently that of the womb, has no influence upon the genuine uterine souffle, the other, on the contrary, is modified and may even cease altogether."

Dr. D. alludes, also, to the occasional transmission of blowing sounds which originate from the heart itself of the mother; but no attentive auscultator can ever confound these with the souffle of pregnancy; for the force or distinctness of the former always increases as we recede from the uterine region towards the cardiac, while that of the latter is diametrically opposite in this respect.

As an illustration of the tenth proposition we may adduce the following case.

Dr. Depaul was summoned to attend a young lady, pregnant with her first child; she considered herself to be at the full period. Upon making an examination however, Dr. D. was surprised to find the uterus so little developed; for its fundus scarcely reached beyond the umbilicus. He therefore suspected either that his patient had made some fault in her reckoning, or else that the fœtus had been dead for a length of time. The fresh details now mentioned by the patient seemed to confirm the latter idea. On repeated auscultation, no fœtal tictac was ever discoverable; but a remarkably strong blowing sound, becoming sometimes of a sibilant character, might be heard over the side of the uterus. Dr. D. communicated his opinion to the husband; and the event proved how completely accurate it was. On the discharge of the liquor amnii, which was sanguinolent and fœtid, the fœtus was quickly expelled. It was in a semi-putrid state, and seemed to have been dead for two or three months. The placenta was the seat of old and serious changes.

This case is but one out of a very large number of similar ones, which have occurred in our author's experience.

The only other remark that we have to make respecting the uterine souffle is, that it becomes much less distinct during the presence of a labour pain, and that it will often disappear altogether when the contractions of the uterus are strong, more especially of the *liquor amnii* has already been discharged. Our next enquiry is about the still more important sign, afforded by the transmission of the sounds of the fœtal heart through the uterine and abdominal parietes. The conclusions, which our author has drawn from all his elaborate researches upon this highly interesting point, are these:

1. The discovery of this auscultatory phenomena preceded that of the uterine souffle, and we owe it to M. Mayor.*

---

* While the claim of the eminent surgeon of Geneva to the merit of this discovery cannot be denied, it is nothing but fair to M. Kergaradec to state that he appears to have been entirely ignorant of it when, in December 1821, he read before the Academy of Medicine his important memoir, announcing the existence of a fœtal as well as of a uterine sound in the latter months of pregnancy.

2. Although there has never been any dispute as to its seat, and as therefore the name given to it is not of much consequence, it seems preferable to adopt that of the double cardiac pulsation (*doubles battements du cœur de l'enfant*).

3. Resembling in some degree the tictac of a watch, it consists of two pulsations that are quite distinct, and, in general, without the admixture of any blowing sound or murmur.

4. Nevertheless, a blowing or a rubbing sound may accompany it; but such an occurrence is rare, nor does it at all indicate the state of health of the child.

5. The double fœtal pulsation may often be heard earlier than is usually supposed. It is frequently discoverable by the end of the fourth month, and occasionally at three months and a half, or even earlier. I have adduced facts which incontestably prove that it may be heard at the end of the 12th, and even of the 11th, week of pregnancy. (This is however of rare occurrence).

6. The absence of the fœtal pulsations is an exceptional occurrence in the three last months* of gestation, unless indeed the fœtus has ceased to live. They were absent, or could not be heard, in 8 only out of 906 women who had advanced to this period.

7. The point of the uterus where they are best heard varies with the period of pregnancy, and still more according to the situation of the child.

8. The spot, which corresponds to the heart, is that which transmits them most forcibly; but, starting from this spot as from a centre, they may be perceived over a greater or less extent; and sometimes they exist, with a variable intensity, over every part of the uterus that is accessible to the stethoscope.

9. In the normal condition, their frequency always exceeds that of the maternal circulation.

10. This frequency is nearly the same at different stages of gestation; it is therefore an error to state that it goes on diminishing in proportion as the pregnancy advances to its completion.

11. The uterine contractions have an obvious, but usually only a very transitory, influence upon the fœtal pulsations.

12. Their force becomes greater as pregnancy advances; but a good deal in this respect depends upon the circumstances of each case.

13. Moral emotions experienced by the mother have no direct influence on the life of the fœtus.

14. Disturbances of the maternal circulation act only consecutively on the fœtal circulation.

15. In my opinion, it is impossible to confound the double pulsations of the fœtus with any other sound which may be heard in the abdomen of a pregnant woman.

16. Their presence certifies not only the existence of pregnancy, but also the life of the fœtus.

---

* In several passages of our author's work it is stated that, in the latter half, or four months and a half, of pregnancy, the absence (or inability to detect the presence) of the fœtal tictac is quite the exception.

17. They may be discovered at a period of pregnancy, when all the other modifications or phenomena lead to but a mere probability.

18. Their absence, ascertained upon several occasions by an experienced observer, will never mislead; and, in every case, this circumstance has a value above that of all the other signs which are usually regarded as indicative of the death of the fœtus.

19. Two double pulsations, distinct and not isochronous, warrant with certainty the diagnosis of a twin-pregnancy.

20. If three such pulsations, each having a peculiar rhythm, were present, it might be possible to recognize a triple pregnancy.

21. The discovery of a fœtal circulation, when at the same time it is known that the uterus cannot contain the product of conception, would suggest the existence of an extra-uterine pregnancy. But, as yet, this is a mere matter of rational conjecture.

22. It is undeniable that, in an immense majority of cases, provided the pregancy be sufficiently advanced, it is quite possible to ascertain with precision how the child lies in reference to the inlet of the pelvis, and the relations of its different parts both with the inlet and with the walls of the uterus.

23. For this purpose, it is not sufficient to make out with certainty the existence of the double pulsations at any one point of the uterus; the exploration must be general, for it is necessary to ascertain what I have called the *summum* of their intensity.

24. In order that the diagnosis, founded on stethoscopic results, may have all the value that can be desired, it is proper that the examination be made, or repeated, at the time when an opinion is given; for it is not impossible, though unquestionably rare, that the fœtus when well developed may, by its own proper movements, modify its position and even its presentation.

25. As it is necessary, in ascertaining the progress of labour, that an examination be every now and then made by the vagina, so, but in a yet higher degree, it is indispensable, as respects the child, to discover at short intervals the state of its circulation.

26. The modifications, which may occur in the double pulsations, ought to be studied with attention; for, when they exceed certain limits, the life of the fœtus will be found to be compromised.

27. These modifications may be only transitory; and then, either they may be sufficient to produce a hurtful influence on the life of the child, and delivery alone can save it; or they may have created a merely temporary distress, which will speedily pass away and not require the interference of art.

28. However this may be, nothing will better enable the physician to appreciate these different conditions than the state of the fœtal circulation.

29. The changes or modifications, which indicate something serious, consist more especially in an irregularity of the pulsations, and in a marked diminution of their frequency and force.

30. Excessive acceleration never indicates any thing amiss; the beats may exceed 200 in a minute, and the child remain quite healthy.

31. The value of such-like facts will be appreciated when the choice of an operation has become necessary—a choice that must be founded on the combined interests of mother and child.

32. In the same measure as the discovery of the uterine souffle is of comparatively little value in practice, so that of the fœtal cardiac pulsations possesses very great importance: no other sign or phenomenon can replace it as a means of exact diagnosis.

On the subject of the seventh of these propositions, we find the following remarks:—

"As the double sound or tictac has its seat in the heart of the fœtus, which, in the early months of pregnancy, enjoys a great mobility within the uterus, we can readily understand how it may be heard at very different spots in the hypogastric region at different times. Moreover, the considerable extent, over which it may be perceived, must serve to produce considerable differences in this respect. It may be said in a general way, that it is in the part of the organ which corresponds to the heart of the child that it will be found, and that from this spot it radiates—diminishing in intensity—over a space of from two to four inches square. In some cases, it extends over a large space at the full term of pregnancy; and it is not impossible but that it may be detected at every point of the uterine globe that is accessible to the stethoscope. The following, however, may be taken as the usual state of things. When it is first perceptible from the twelfth to the sixteenth week, it is by the fundus of the uterus, which then begins to exceed the pelvic inlet and can alone be explored, that it is transmitted. Most usually, it is by no means a matter of indifference in what way the pressure with the stethoscope is made. It is almost always necessary to give it a vertical direction, *i. e.* parallel with the axis of the uterus itself. As a matter of course, the bladder should always be quite empty at the moment of exploration.

"In proportion as the uterus rises above the pelvis, the preceding considerations are less frequently applicable, and then it is over one or other of the lateral regions of the organ that we may expect to find the sound; although it may be perceptible also upon the median region. According to my experience, however, this is of much less frequent occurrence than at an earlier stage of pregnancy. The explanation of the difference is probably this. From the fifth to the sixth month, the size of the fœtus is much more considerable, and the quantity of *liquor amnii* is proportionately less; hence there is more fixedness in the relations, and more facility to keep the uterine parietes in contact with certain regions of the fœtal ovoid. We shall presently see that these are not all equally capable of transmitting the cardiac sound.

"But it is especially in the three last months of gestation that the influence of the conditions just mentioned becomes evident; and this in such a marked manner, that, if the exact position of the fœtus were previously known, one might with almost complete certainty indicate the region upon which the stethoscope should be placed. The results of an extended series of observations warrant me in asserting that, in a large majority of cases, the double sound will be found along the trajet of a line which, extending from the left antero-superior spine, would terminate in the umbilicus; that, much less frequently, it will be found over the correspondent points on the right side; and that, much more rarely still, it will be found above the umbilicus, sometimes on the left and at other times on the right side. It is at this period also that it may extend from its point of departure over a considerable space, and that, in some instances, it is even discoverable over every accessible point of the uterus."

Dr. Depaul has some very interesting observations on the independence of the fœtal and maternal circulations, that well deserve to be attended to; but we must refer our readers to his work for particulars. We cannot, however, pass them over altogether without noticing briefly a very interesting case, related by our author in illustration of this point.

A young woman, in the advanced stage of pregnancy, fell off the chair on which she was sitting at work, and, after two or three deep-fetched respirations, expired. In the course of about four minutes from the time of the fatal event, Dr. Campbell, an *interne* of the Maternity, ascertained that life was quite extinct: there was no respiratory murmur to he heard, nor any pulsation of the heart. In the report of the case, communicated by this gentleman to our author, the following particulars are given :—

"Immediately after the death of the mother, the presence of a living child in the uterus was ascertained; the pulsations of the fœtal heart were distinctly heard, their number being from 120 to 140 in the minute.

"While I was examining the chest of the mother, my hand, resting on the abdomen distended by the uterus, felt some active movements: the shock was pretty strong, brusque, and repeated at short intervals. Auscultation enabled me at this time—about six minutes after the death of the woman—to detect the sounds of the fœtal heart in the left iliac fossa: they were regular, tolerably strong, and did not exceed about 100 in the course of the minute. Two or three minutes subsequently, they had fallen down to between 60 and 80.

"The circumstance of the rapid diminution of the fœtal pulsations, added to the brusque character of the active movements of the fœtus (a truly pathognomonic character of compromised vitality) on the one hand, and, on the other hand, the certainty of its almost immediate death if something were not done without delay, induced us to have recourse to the Cæsarian section, which was commenced ten minutes at least after the death of the mother. The child, although living when extracted, did not make an inspiration until 25 or 30 minutes had been spent in insufflation by means of a laryngeal tube. It was only when a large quantity of amniotic fluid had been rejected by the mouth, nose, and the tube itself, that it began to utter a few feeble moans; nor was it relieved from the state of general cyanosis and insensibility, before a small quantity of blood was allowed to flow from the divided end of the cord. It is now fifty days old, and seems to thrive very well."

From the consideration of this and such-like cases, as well as from other circumstances (of which one of the most remarkable is the fact that, while the inhalation of æther has the uniform effect of accelerating the pulse of the mother, it scarcely, if at all, affects that of the fœtus), Dr. Depaul is of opinion that it may be fairly regarded as proved "that there is no direct connexion between the maternal and the fœtal circulation,—that derangements of the former will sometimes persist for a considerable time without reacting in any manner upon the latter;—that nevertheless, when these derangements are of a nature to produce profound modifications, inappreciable though these may be by their physical characters, in the blood of the mother, they exert an influence, after a variable time, upon the fœtus, and this influence is evidenced first by an increased frequency of the fœtal pulsations, and then by a diminution of their frequency, which is a sign of much more serious import;—lastly, that the moral emotions of the mother can act upon the child only consecutively, and through the intervention of the blood."

In reference to the 18th proposition, the following details will be read with interest. In 17 out of 26 cases, in which the period of pregnancy was from the end of the third to that of the fifth month, and in which our author could detect no fœtal pulsation, the result proved the correctness of his diagnosis. In the majority of the remaining cases, the pregnancy had not exceeded the fourth month. As to the cases, he adds, in which he had once heard the sound, the negative result obtained subsequently never misled him : the death of the fœtus was always demonstrated ultimately.

Out of 67 cases, in which the women were in the four last months of gestation, the absence of the fœtal pulsations, taken as the evidence of the death of the child, disappointed our author's diagnosis only on three occasions. He very properly reminds his readers that, in order that the examination may be regarded as conclusive, not only must the physician have had very considerable experience, but the exploration also must have been repeated on several occasions on two or three different days.

There is a case reported by M. Naegele, in the Medicinische Annalen for last year, that bears upon the proposition 20 : it is entitled, *Results furnished by Auscultation in a case of Triplets.* A woman, in labour with her first child, was ausculted by M. N., who found in the left hypogastric region the double beat of the fœtal heart, as well as a simple blowing sound of the umbilical cord, and in the right hypogastric region two other double pulsations. Upon two observers examining with the stethoscope, one on each side, at the same time, it was found that the beats of the fœtal heart and of the cord were rather more frequent on the right than on the left side (152 to 144). Subsequently, after the right fœtus had been in brisk motion, the heart was found to give 160 beats, and the cord only 144 ; there was thus a difference of 16 beats in the minute. After the delivery of a first child, auscultation was again practised; and still the pulsations of two hearts were heard distinctly, the one set in the left hypochondriac, and the other in the right umbilical, region. The head of the child, which next presented in the vagina, was found to be in the second position ; consequently the cardiac pulsation on the right side belonged to it. After its expulsion, the fœtal pulsations, indicating the presence of a third child, were still readily to be heard : this child was born without the assistance of art.

Although the presence of three children was only made out after the birth of the first one, the case still serves to shew how much information auscultatory means are capable of affording. It should be mentioned that there was no appreciable difference in the uterine souffle, either as respects intensity or the extent of space over which it was perceptible.

Had our space permitted, we might have felt inclined to have given, at some length, an abstract of Dr. Depaul's observations as to the possibility of ascertaining the position of the fœtus in the womb by attending to the exact spot where the tictac is loudest, and where, consequently, the heart of the child is situated. All that we can do is to make one or two very short extracts from his elaborate narrative.

After alluding to the inapplicability of the test in question in the first five or six months of gestation, in consequence not only of the insufficient development of the uterus, but also of the small dimensions of the fœtus and its extreme mobility in the *liquor amnii*, he goes on to say :—

"But at seven months the relations of the child to the uterus are much less variable; the great axis of the child has, from this time, such dimensions as rarely permit one of its extremities from taking the place of the other. The movements in the line of this axis are somewhat more frequent and more easily understood; but these become more rare as the full period of gestation approaches, so that at the end of the eighth month they are mere exceptions which are very seldom met with."

Still, however, cases do occur, where the child seems to have made a complete somerset within the uterus, at an advanced period of pregnancy. Allowance must therefore be made for such occasional occurrences.

To understand the following remarks, let the abdominal uterine globe be supposed to be divided into four parts by a transverse line passing a little below the umbilicus, and crossing a perpendicular one drawn from the epigastrium to the pubes.

"When the fœtal pulsation is found in the left inferior region, the presentation will be that of the head, and the back of the child will be turned towards the left side; when in the right inferior region, the presentation will be the same, and the back of the fœtus be turned to the right; when, on the contrary, the sound is heard in the left superior region, the pelvic extremity will be the part most depending, the back being turned to the left; when in the right superior region, the position will be the same, with the exception of the back being directed to the right side."

With the view of rendering his observations on this hitherto little-explored subject of obstetrical enquiry better understood, our author has introduced several illustrative wood-cuts representing the relations of the fœtus in different positions in the pelvis.

We need scarcely say that we think very highly of Dr. Depaul's volume, and that we recommend it in the strongest terms to the attention of the profession in this country.

